# The complete mitochondrial genome of the half-fin anchovy, *Setipinna tenuifilis* (Valenciennes, 1848)

**DOI:** 10.1080/23802359.2021.1962753

**Published:** 2021-08-16

**Authors:** Yong Liu, Yibang Wang, Hui Jia, Hui Zhang, Weiwei Xian

**Affiliations:** a Shandong Museum, Jinan, PR China; b CAS Key Laboratory of Marine Ecology and Environmental Sciences, Institute of Oceanology, Chinese Academy of Sciences, Qingdao, PR China; c University of Chinese Academy of Sciences, Beijing, PR China; d Qingdao Agricultural University, Qingdao, PR China; e Laboratory for Marine Ecology and Environmental Science, Qingdao National Laboratory for Marine Science and Technology, Qingdao, PR China; f Center for Ocean Mega-Science, Chinese Academy of Sciences, Qingdao, PR China

**Keywords:** Mitochondrial genome, *Setipinna tenuifilis*, next-generation sequencing, phylogenetic

## Abstract

The complete mitochondrial genome of the half-fin anchovy, *Setipinna tenuifilis* collected from Yellow and Bohai Seas was determined by next-generation sequencing. The mitogenome is a circular molecule 16,668 bp in length, including the typical structure of 13 protein-coding genes, two ribosomal RNA genes, 22 transfer RNA genes, and a control region. The termination-associated sequence (TAS), central conserved sequence block (CSB) and CSB are detected in the control region. The gene contents of the mitogenome are identical to those observed in most bony fishes.

The half-fin anchovy is an ecologically and economically important Engraulidae species, widely distributed in Pacific Ocean and along most of China’s coastline, especially in the Bohai Sea. The Yellow and Bohai Seas are important spawning and feeding grounds for biological resources, including half-fin anchovy. Unfortunately, half-fin anchovy has been decreasing gradually due to excessive fishing pressure (Wang et al. 2020). Currently, research on half-fin anchovy mainly focuses on its distribution, population structure, and biology (Cai et al. 2014). However, the half-fin anchovy has not received much attention in population genetic studies in the area of Bohai and Yellow Seas.

Next-generation sequencing (NGS) has improved the speed and throughput capacities of DNA sequencing, thus greatly reducing the overall cost of sequencing (Zhang et al. 2011). The emergence of NGS has promoted the characterization of mitochondrial genomes in various species (Li et al. 2019), which can be used to provide insight into population genetics and evolutionary history of species (Zhang and Xian 2016). In the present study, we used NGS methods to sequence the complete mitogenome of *Setipinna tenuifilis* using DNA extracted from muscle tissue collected in September 2019 from the Bohai and Yellow Seas (122.17°E, 37.53°N). The specimen was deposited in CAS Key Laboratory of Marine Ecology and Environmental Sciences, Institute of Oceanology, Chinese Academy of Sciences, and its catalog number was ST 113-1. We established the phylogenetic relationship among the half-fin anchovy and the other 13 closely related Engraulidae species, aimed to determine the phylogenetic status of the half-fin anchovy, although the two previously *Setipinna tenuifilis* mitogenomes (MH037012.1 and AP017950.1) were already published (Lavoué et al. 2017; Fan et al. 2018).

This complete mitogenome of *Setipinna tenuifilis* was 16,668 bp in length (GenBank accession no. MT764773), within the range of other teleost mitogenomes. However, the mitogenomes from other specimens collected from Shanghai (MH037012) (Fan et al. 2018) and Taiwan (AP017950) (Lavoué et al. 2017) were 16,215 bp and 16,884 bp, respectively, which differed from this new *S. tenuifilis* mitogenome also from the Northwest Pacific Ocean. As in other vertebrates (Miya et al. 2001), it contained 13 protein-coding genes, two rRNA genes (12S rRNA and 16S rRNA), 22 tRNA genes, and a control region, which was consistent with the published data (Lavoué et al. 2017; Fan et al. 2018). Like other bony fishes, most mitochondrial genes of *S. tenuifilis* were encoded on the H-strand, with only ND6 and eight tRNA genes encoded on the L-strand, which was consistent with the findings of Fan et al. (2018). Two overlapping reading frames, which had not been mentioned in the other studies, were detected on the same strand in six of the 13 protein coding genes (ND1, COI, ND4, ND5, ND6, Cyt b). The ATPase 6 and ATPase 8 overlapped by 10 nucleotides, and ND4 and ND4L shared seven nucleotides. ND5 and ND6 overlapped by four nucleotides on the opposite strand. This was not consistent with the findings of Fan et al., where ATPase 8 and ATPase 6 overlapped by two nucleotides (Fan et al. 2018). ATG was the initiation codon of 12 out of the 13 protein coding genes, while the initiation codon of COI was GTG, which was different from the results of Fan et al. (2018). The 12S and 16S ribosomal RNA genes of *S. tenuifilis* comprised 951 bp and 1652 bp, respectively. Interestingly, the results of the 12S were the same as that of the Shanghai sample, and there were some differences in the 16S (Fan et al. 2018). The 12S rRNA was located between tRNA^Phe^ and tRNA^Val^, and 16S rRNA between tRNA^Val^ and tRNA^Leu^, as observed in other teleost fishes (Gao et al. 2013). The 22 tRNA genes were distributed in the genome and ranged in size from 67 to 75 bp and fold into cloverleaf secondary structures with normal base paring, which could be found in Fan’s study as well (Fan et al. 2018). The major noncoding region in *S. tenuifilis* was located between tRNA^Pro^ and tRNA^Phe^ and was determined to be 389 bp in length, which was different with the samples from Shanghai and the Northwest Pacific, respectively, 1254 bp and 1223 bp (Lavoué et al. 2017; Fan et al. 2018). The termination-associated sequence (TAS), central conserved sequence block (CSB) and CSB were detected in the control region, which was similar to most bony fishes (Zhang et al. 2013).

Phylogenetic analysis (maximum likelihood) was accomplished by Mega X, using the complete sequence of *Setipinna tenuifilis* and the other 13 closely related Engraulidae species, the samples from *Setipinna tenuifilis* included (Figure 1). All three *S. tenuifilis* mitogenomes clustered into a clade, with the specimen collected from the Bohai and Yellow Seas (MT764773) showing greater similarity to the specimen from Shanghai (MH037012), rather than to the other specimen from Taiwan (AP017950). That relationship, combined with the considerable nucleotide sequence difference in the D loop region of the mitogenomes, suggests that *S. tenuifilis* populations in Taiwan may be geographically isolated from *S. tenuifilis* populations in the Yellow and Bohai Seas. It is important to note as well, however, that the mitochondrial genome of the Shanghai specimen (MH037012) is in an unverified state.

**Figure 1.**
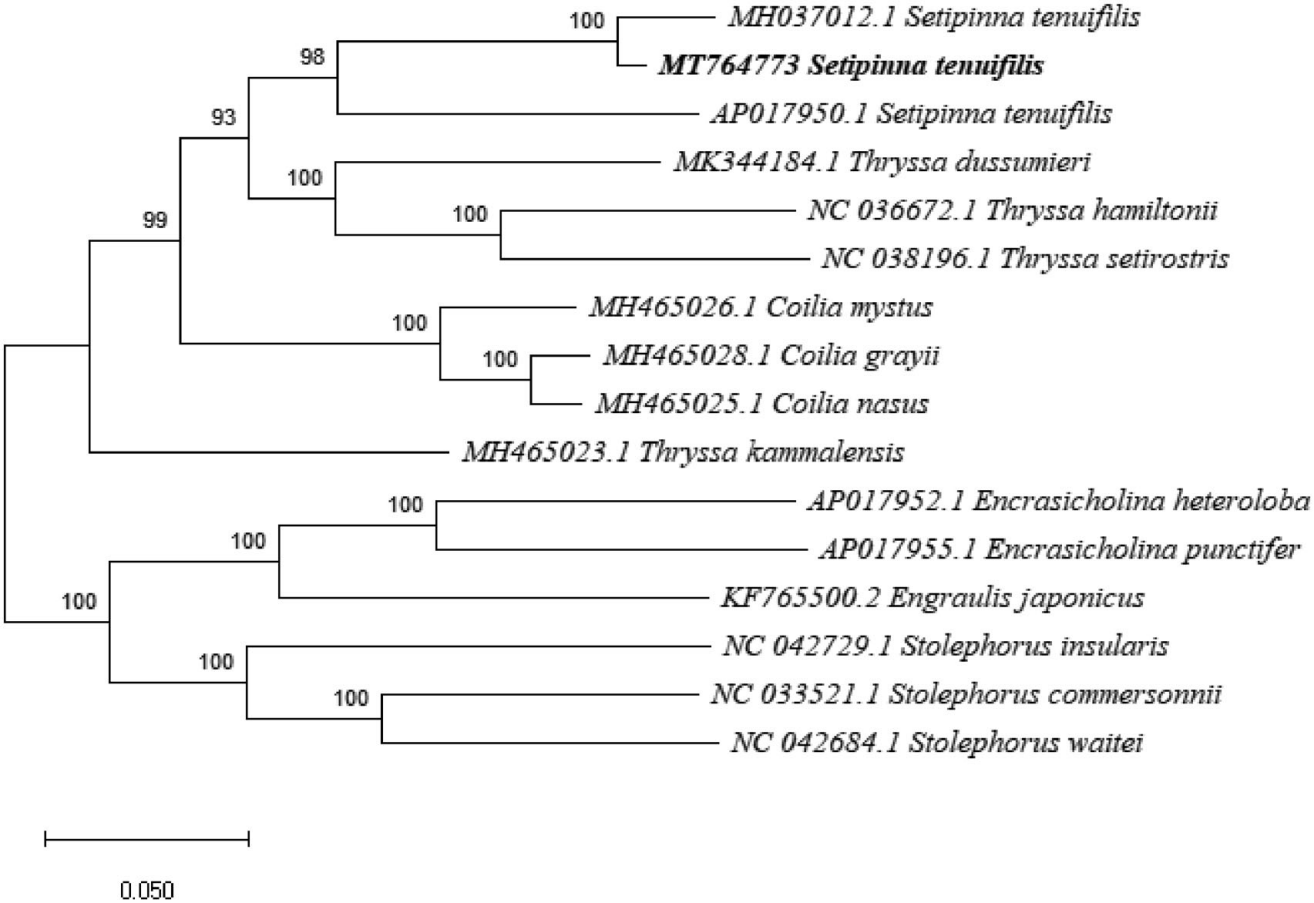
Phylogenetic relationship revealed by ML tree among 14 Engraulidae species based on mitochondrial complete sequence.

## Data Availability

The genome sequence data that support the findings of this study are openly available in GenBank of NCBI at https://www.ncbi.nlm.nih.gov/nuccore/MT764773. Associated BioProject, SRA, and BioSample accession numbers are https://www.ncbi.nlm.nih.gov/bioproject/PRJNA686147, https://www.ncbi.nlm.nih.gov/sra/SRR13269484, and SAMN17108034, respectively.
